# Physicochemical Characteristics and Composition of Three Morphotypes of *Cyperus esculentus* Tubers and Tuber Oils

**DOI:** 10.1155/2015/673547

**Published:** 2015-10-11

**Authors:** Souleymane Bado, Patrice Bazongo, Gouyahali Son, Moe Thida Kyaw, Brian Peter Forster, Stephan Nielen, Anne Mette Lykke, Amadé Ouédraogo, Imaël Henri Nestor Bassolé

**Affiliations:** ^1^Plant Breeding and Genetics Laboratory, Joint FAO/IAEA Division of Nuclear Techniques in Food and Agriculture, International Atomic Energy Agency, Vienna International Centre, P.O. Box 100, 1400 Vienna, Austria; ^2^Life and Earth Sciences Training and Research Unit, University of Ouagadougou, 03 BP 7021, Ouagadougou, Burkina Faso; ^3^Département Mécanisation, Institut de Recherche en Sciences Appliquées et Technologies, 03 BP 7047, Ouagadougou 03, Burkina Faso; ^4^Department of Medical Research (Lower Myanmar), No. 5 Ziwaka Road, Dagon Township, Yangon 11191, Myanmar; ^5^Department of Biosciences, Aarhus University, Vejlsoevej 25, 8600 Silkeborg, Denmark

## Abstract

Tuber characteristics and nutrient composition of three morphotypes of *Cyperus esculentus* tubers and tuber oils were determined. The mean value for length and width of the tuber and one thousand dried tuber weights ranged from 0.98 to 1.31 cm, 0.90 to 1.19 cm, and 598 to 1044 g, respectively. Tubers displayed high level of starch (30.54–33.21 g 100 g^−1^), lipid (24.91–28.94 g 100 g^−1^), and sucrose (17.98–20.39 g 100 g^−1^). The yellow tubers had significantly higher content in lipid compared to black ones. Levels of ascorbic acid, tocopherol, and *β*-carotene of the three morphotypes differed significantly. Yellow ones (morphotypes 1 and 2) were the richest in tocopherol and the poorest in *β*-carotene. Saturated fatty acid content of morphotype 2 was significantly lower than that of morphotypes 1 and 3. Morphotype 3 had the significantly lowest PUFA content compared to morphotypes 1 and 2. Morphotype 1 was found to be richer in Ca, Cu, and Mn contents. Al, Mg, P, S, and Si were most abundant in morphotype 2. Morphotype 3 had the highest content of Cl, K, and Zn.

## 1. Introduction

Cyperaceae is a family of monocotyledonous graminoid flowering plants known as sedges, which superficially resemble grasses or rushes. About 5,500 species have been described in the family [1] including* Cyperus esculentus*.* Cyperus esculentus* provides edible tubers commonly called tigernut, chufa sedge, nut grass, yellow nutsedge, tigernut sedge, or earth almond. Tigernut is a perennial crop cultivated particularly in tropical and subtropical areas worldwide and extensively in Africa, Asia, and some European countries for their sweetish tubers. In Africa, tigernut is mostly cultivated in the west, Ivory Coast, Ghana, Mali, Niger, Nigeria, Senegal and Togo where they are used primarily uncooked as a side dish [2]. The tubers are used fresh as a vegetable or dried as a sweet snack. They are also grinded into flour and used as a thickener, for bread and cakes or mixed with water as drink. The tubers are often considered as “health” food because they have excellent nutritional properties and prevent heart diseases and thrombosis. Tigernut is known to activate blood circulation, to reduce risk of colon cancer and diabetes, and to favor weight loss [3]. Tigernut is also known to have aphrodisiac, carminative, diuretic, emmenagogue, stimulant, and tonic effects and even some medicinal uses such as treatment of flatulence, indigestion, diarrhea, dysentery, and excessive thirst [4]. Tigernut is used as livestock food and is in southern USA ranked among the top 10 most important waterfowl foods [5]. Tigernut flour is a rich source of carbohydrate, oil, and some useful mineral elements such as iron and calcium which are necessary for body growth and development [6, 7]. Three varieties have been reported on the basis of their color, namely, yellow, black, and brown varieties [8]. Tigernut was reported to be rich in carbohydrates, dietary fiber, lipids, and oleic acid [3, 9]. Despite its great potentialities the tigernut remains an underutilized plant [7]. Most of the studies focused on the yellow variety while very little information exists on the physical characteristics of tigernut tubers. A better understanding of morphological parameters of the tigernut tubers as well as their link to the nutrition composition will help to identify valuable varieties and promote their use. So, this crop could contribute to the poverty alleviation among vulnerable populations, particularly rural women, in Western Africa. The aim of this study was to determine the physical traits as well as the chemical characteristics of the tubers from the three morphotypes of tigernut grown in Burkina Faso.

## 2. Material and Methods

### 2.1. Plant Material

Tubers of* Cyperus esculentus* L. were sampled in January and February 2007–2009, in 5 villages located in western and southwestern Burkina Faso: Loropéni (10°18′N, 3°32′W), Mangodara (9°54′N, 4°21′W), Ouéléni (10°51′N, 5°21′W), Tangora (10°38′N, 4°45′W), and Tiéfora (10°38′N, 4°33′W). Five kilograms of tubers was collected in each village, immediately hand-sorted to eliminate damaged ones, and taken to the laboratory. Prior to any analysis, the samples were washed with distilled water, drained, and air-dried. Each village sample was split into two parts; one part was finely ground with a Moulinex grinder robot (GT550, Zurich, Switzerland). Both parts were packing in an airtight container and stored at −18°C until analysis.

### 2.2. Analytical Methods

#### 2.2.1. Physical Analysis

To determine the mean length and width of the tubers, 100 tubers were per village randomly picked and their two linear dimensions were measured using a Vernier caliper with an accuracy of 0.01 mm (Canon Instruments, Japan). The thousand dried tubers weight (TSW) was obtained by counting 1000 dried tubers and weighted on an electronic balance to 0.001 g accuracy (Ohaus, USA). The variation in tubers size and color was used to classify the tigernut into different morphotypes.

#### 2.2.2. Chemical Analysis

The official methods of the Association of Official Analytical Chemists [10] were used to determine moisture, protein, crude oil, and ash contents of the tubers. Moisture (g water 100 g^−1^ sample) was determined by drying a 3 g ground sample at 105°C to constant weight. Nitrogen content was determined by using the Kjeldahl method and multiplied by a factor of 6.25 to determine the crude protein content (g protein 100 g^−1^ sample). Crude fat (g fat 100 g^−1^ sample) was obtained by exhaustively extracting 5.0 g of each sample in a Soxhlet apparatus using petroleum ether (boiling point range 40–60°C) as the extractant. Mineralization was performed on 3 g samples by combustion in a muffle furnace at 550°C for 8 h (g ash 100 g^−1^ sample) (AOAC 920.39C). Carbohydrate content was estimated by difference of mean values: 100 − (sum of percentages of ash, protein, and lipids) [11].

#### 2.2.3. Starch and Sugar Analysis

AOAC method 996.11 was used to determine starch content of* Cyperus esculentus* tuber flours. The assay consisted of using thermostable alpha-amylase and amyloglucosidase to enzymatically hydrolyze starch into glucose that was then quantified with a spectrophotometer (*μ*Quant, Bioteck Instruments Inc, USA). Glucose, sucrose fructose, and maltose were analyzed by HPLC according to the AOAC Official Method 982.14 [12]. Samples for HPLC sugars analysis were prepared by homogenizing 0.3 g of* Cyperus esculentus* flour in 3 mL distilled water and 7 mL 95% alcohol and shaken before being centrifuged at 10 000 rpm for 20 min. The clear supernatant was filtered through 0.45 *μ*m filter and degasified before analysis by HPLC. Filtered solution (20 *μ*L) was injected into HPLC 1100 Series (Agilent, Waldbronn, Germany) equipped with a G1362A refractive index detector. Sugars were separated using a commercially packed with Zobax-NH_2_ column (250 × 4.6 mm (Dupont, Wilmington, DE, USA)) with a particle size of 5 *μ*m and thermostatized at 30°C. The filtered and degasified mixture of acetonitrile/water (80/20) was used as mobile phase at a flow rate of 1 mL/min for 30 min run time [13]. The sugars peaks were identified by comparing their retention times with individual standard sucrose, maltose, glucose, and fructose approximately 99% pure (Sigma-Aldrich, Steinheim, Germany) and the chromatograms analyzed using the Agilent Technologies Chemstation Software.

#### 2.2.4. Vitamin Analysis

Vitamin C was determined in tubers as previously described [14, 15]. An aliquot of 25 g of tigernut was added to 25 mL of a solution containing 45 g/L metaphosphoric acid and 7.2 g/L of DL-1,4-dithiotreitol (DTT). The mixture was homogenized and centrifuged at 22,100 g for 15 min at 4°C. The supernatant was vacuum-filtered through Whatman no. 1 filter. Prior to HPLC analysis, the vacuum-filtered samples (10 mL) were passed through a Millipore 0.45 *μ*m membrane. Then, 20 *μ*L was injected into a HPLC system fitted with a reversed-phase column, C18 Spherisorb ODS2 (5 *μ*m) stainless-steel column (4.6 mm × 250 cm). The mobile phase was a 0.01% sulphuric acid solution adjusted to a pH of 2.6, at a flow rate of 1 mL/min at room temperature. Detection was performed at 245 nm with 486 Absorbance Detector (Waters, Milford, MA). Vitamin C was quantified through a calibration curve built with ACS grade ascorbic acid (>99% pure, Sigma-Aldrich, Steinheim, Germany) pure standards in the range of 0.2–50 *μ*g/mL.

To determine vitamin E (*α*-tocopherol) and *β*-carotene, approximately 5 g of ground samples were extracted with 50.0 mL of hexane. The mixture was then vortexed for 5 min and filtered using 0.2 *μ*m pore size PTFE membrane. The filtered hexane fraction was directly injected into RP-HPLC system for *β*-carotene and vitamin E analysis [16]. The RP-HPLC system (Shimadzu) consisted of an autosampler and column oven equipped with Inertsil ODS-3V (250 × 4.6 mm, 5 *μ*m) reversed-phase column. For *β*-carotene analysis, mobile phase was acetonitrile (6 : 4, v/v, containing 0.05: BHA as antioxidant) (eluent A) and MeOH (eluent B). The following gradient was used: initial condition was 70% (A) and 30% (B) for 5 min, followed by 80% (A) and 20% (B) for 5 min, at a flow rate of 1.5 mL/min. Elution was monitored using a photodiode-array detector at 472 nm [17]. For vitamin E content, methanol mobile phase was used at a flow rate of 1.0 mL/min. The *α*-tocopherol was detected by a Shimadzu SPD-10A (UV/VIS) detector (292 nm wavelength). Standards of *β*-carotene (≥97.0% purity, Sigma-Aldrich, Steinheim, Germany) and DL-*α*-tocopherol (≥96% purity, Sigma-Aldrich, Steinheim, Germany) ranging from 0.5 to 6.0 *μ*g/mL and from 0.02 to 1.0 *μ*g/mL were used for calibration.

#### 2.2.5. Tuber Oil Fatty Acids Analysis

Fatty acid methyl esters were determined according to International Union of Pure and Applied Chemistry (IUPAC) method II.D.19 [18]. On hundred milligrams of extracted oils was saponified in a volumetric flask, with 1.2 mL of 0.5 M KOH in MeOH by heating and stirring under reflux for 5 min. After saponification oils were esterified by adding 1.2 mL 20% borontrifluoride through condenser and boiled for 2 min and then the flask was moved from the magnetic stirrer and fatty acid methyl esters were extracted by adding 1 mL of n-hexane. Saturated NaCl solution was added until the n-hexane is in the neck of volumetric flask, mixed carefully, flipped once or twice, and let settle for about 30 min. After separation the n-hexane phase was transferred to a vial for fatty acid methyl esters analysis. Gas chromatography (GC) of fatty acid methyl esters was performed using a Perkin Elmer GC-autosystem XL with a programmable temperature vaporizer (PTV) split-injector and a flame ionization detector (FID). Helium was used as carrier gas. The column temperature was initially maintained at 100°C for 2 min and then raised by 5°C/min to 225°C and finally held at 225°C for 16 min. The injection volume was 0.2 *μ*L with a 1 : 100 split. The PTV injector was initially maintained at 50°C and immediately after the injection raised to 270°C. The FID was kept at 250°C. The capillary column employed was CP Sil 88 (Chrompak, Varian Instruments, Walnut Creek, CA; 50.0 m × 0.25 mm and 0.2 *μ*m film thickness). The peaks were identified by comparing retention times with authentic fatty acid methyl esters. Quantification was based on the area under each fatty acid peak as compared to the total area of all fatty acid peaks.

#### 2.2.6. Mineral Composition of Powered Tubers

The elements, Mg, P, Cr, Fe, Mn, Cu, Zn, Sr, Ca, K, and Cd, in digests were measured using an atomic absorption spectrophotometer (Analyst 800, Perkin-Elmer) and/or a coupled plasma mass Spectrophotometer (ELAN DRCII Axial Field Technology, Perkin-Elmer). About 0.2 g of powered tuber was digested with 3 mL of HNO_3_ (65%) and 0.5 mL of H_2_O_2_ (30%) in a closed vessel microwave digestion system (MLS-ETHOS plus) and diluted to 50 mL with Millipore water. Digestion conditions for the microwave system were applied as follows: 2 min for 250 W, 2 min for 0 W, 6 min for 250 W, 5 min for 400 W, 8 min for 550 W, and vent for 8 min. A blank digest was carried out in the same way. Al, Si, S, and Cl were analysis by polarized Energy Dispersive X Ray Fluorescent (EDXRF), Spectro X-LAB 2000. Prior to analysis, 4 g of ground dried samples triplicate was pelleted by 5 tons using SpectroPess (Chemplex Industries, Inc.) and then pellets were analyzed using different excitation conditions with an EDXRF spectrometer [19]. Standard Reference Material 1568a rice flour was obtained from National Institute of Standards and Technology, Gaithersburg, USA, and was used as food reference material to evaluate the analytical methods.

### 2.3. Statistical Analysis

All samples were tested at least in duplicate in each analytical technique. The values of different parameters were expressed as the mean ± standard deviation. Comparison of means was performed by one-way analysis of variance (ANOVA) followed by Wilcoxon's multiple comparison tests. Principal component analysis (PCA) was performed to compare the physical and chemical data of 3 morphotypes of* Cyperus esculentus* tubers. PCA was carried out using the 33 physical and chemical variables which differed significantly between morphotypes. Principal component analysis (PCA) is used in exploratory analysis. It gives graphical representations of intersample and intervariable relationships and provides a way to reduce the complexity of the data. Statistical significance was set at the 5% level of probability using JMP In 5.1 software (SAS Institute, Cary, NC, USA).

## 3. Results and Discussion

### 3.1. Morphological Variants

**Figure 1:**
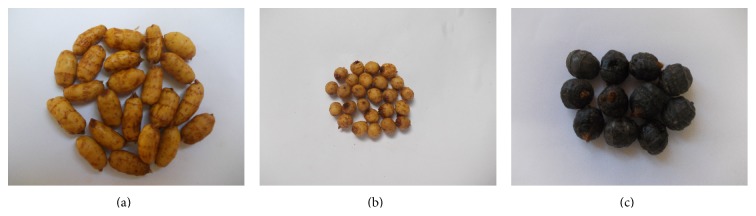
Morphotypes of* Cyperus esculentus* grown in Burkina Faso. (a) Morphotype 1: big size and yellow color. (b) Morphotype 2: small size and yellow color. (c) Morphotype 3: big size and black color.

Tubers from five collection sites were grouped into three morphological variants on the basis of their color (yellow or black) and size (big or small tuber). Then three variants were identified: (1) yellow and big (morphotype 1), yellow and small (morphotype 2), and black and big (morphotype 3) (Table 1). Thus, tuber samples from Mangodara and Tiéfora were classified as morphotype 1, those from Loropéni and Ouéléni as morphotype 2, and those from Tangora as morphotype 3 (Figure 1).

### 3.2. Physical Characteristics

The tuber characteristics of the three morphotypes of* Cyperus esculentus* are shown in Table 1. The moisture content was not significantly different (*p* > 0.05) among the three morphotypes. Lengths were ranged from 0.98 ± 0.06 to 1.31 ± 0.06 cm. Morphotype 2 tubers were significantly shorter than those of morphotypes 1 and 3. Morphotypes 1 and 3 had slightly bigger tubers than approximate average length (0.63 to 1.21 cm) found for tigernuts from other countries [5]. The width of the tuber and one thousand dried tuber weights varied from 0.90 ± 0.08 to 1.19 ± 0.05 cm and from 598.00 ± 115.00 to 1044.00 ± 394.60 g, respectively. The tuber width of morphotype 2 was significantly lower than that of morphotype 3. Both morphotypes 1 and 3 had higher one-thousand-tuber weight than morphotype 2. The one-thousand-tuber weight seems to be more influenced by tuber width than length. One thousand weights of investigated tubers were far higher than those obtained for brown tubers by Coşkuner et al. [5] that showed how genetic diverse is* C. esculentus* cultivated around the world.

### 3.3. Proximate Composition

Crude oil contents of the three morphotypes varied from 24.91 ± 0.94 to 28.94 ± 0.37 g 100 g^−1^ of dry weight (DW). Crude oil content was higher in morphotypes 2 followed by morphotype 1, with morphotype 3 as the lowest (Table 1).

Crude oil content of the three morphotypes reported in this paper is lower than those reported for black and brown tubers from Cameroon [20]. However, the lipid content values are similar to those of white tubers [21] and higher than the content of tigernut genotype from Spain reported by Alegría-Torán and Farré-Rovira [22]. The data reported indicate that the lipid content of tigernut is influenced by genetic material and geographical location. Protein levels in the three morphotypes ranged from 3.3 ± 0.26 to 4.33 ± 0.6 g 100 g^−1^. The morphotype 2 protein content was significantly higher than morphotype 3. The levels of protein are not related to either colour or size. The protein content for the three morphotypes from Burkina Faso was very low compared to tubers from Cameroon [20], Nigeria [23], and Turkey [5]. The ash content of the three morphotypes was ranged between 1.81 ± 0.24 and 2.21 ± 0.39 g 100 g^−1^. Morphotype 3 had significantly higher ash content than morphotype 2. No significant difference was found between morphotype 1 and the two other ones. The ash content of the three morphotypes is lower than those reported for black, brown, and yellow tubers [5, 20, 21]. Morphotypes 1 and 3 had similar carbohydrate content which was higher than that of morphotype 2. Tubers from Burkina Faso are richer in carbohydrates than those from Nigeria [7] and Spain [22].

### 3.4. Starch and Other Carbohydrate Contents

Starch, sucrose, fructose, and glucose contents of three morphotypes are reported in Table 2.

The starch content of the three morphotypes ranged from 30.54 ± 2.75 to 33.21 ± 1.1 g 100 g^−1^. It appeared that starch content was not significantly different among morphotypes. However, the values for the three morphotypes were slightly higher than those reported by Coşkuner et al. [5] and similar to data of Linssen et al. [24]. The tigernuts from Burkina Faso displayed 1.6 ± 0.69 to 3.59 ± 0.72 g 100 g^−1^ of fructose with no significant difference among morphotypes. The sucrose and glucose contents of the three morphotypes ranged from 17.98 ± 1.03 to 20.39 ± 1.15 g 100 g^−1^ and from 0 to 6.79 ± 1.34 g 100 g^−1^, respectively. Morphotype 3 had significantly higher sucrose than morphotype 1 and morphotype 2, while glucose content was not detected. The sucrose content was within the range previously reported [5, 25]. Karacali [26] reported that the amount and composition of sugars vary according to fruit species, varieties, and ecological conditions, and technical and cultural practices affect the flavour. In addition, irrigation, harvest time, and storage conditions also affect the sugar composition of almond kernel [27–29]. Regarding the taste of sugars, sucrose is sweeter than glucose, and fructose is sweeter than sucrose [30]. Balta et al. [31] reported a positive correlation between glucose and fructose contents and the sweet taste of almond. Yellow morphotypes (morphotypes 1 and 2) had higher glucose and fructose contents than black ones; they should be sweeter.

### 3.5. Vitamin Contents

The ascorbic acid, tocopherol, and *β*-carotene contents of the three morphotypes are shown in Table 3. Vitamins contents of the three morphotypes differed significantly. The ascorbic acid levels varied from 5.48 ± 1.05 to 26.78 ± 2.51 mg 100 g^−1^ and were within the usual range for tubers and lower than that of nuts [32, 33]. The highest content of ascorbic acid was recorded with morphotype 2, followed by morphotypes 3 and 1.

Tocopherol content of three morphotypes ranged from 149.86 ± 1.94 to 270.56 ± 8.33 *μ*g 100 g^−1^. The morphotype 2 tocopherol content was significantly higher than that of morphotype 1, which was also significantly higher than that of morphotype 3. The tocopherol content obtained in this study is lower than that reported in tigernut oil from Ghana [9].

*β*-Carotene content of three morphotypes varied from 6.13 ± 0.62 to 10.05 ± 1.79 *μ*g 100 g^−1^. Morphotypes 2 and 3 had the lowest and the highest content, respectively. Burmeister et al. [34] reported higher *β*-carotene content compared to Burkinabe tubers.

### 3.6. Fatty Acid Composition

Oils of the three morphotype tubers contained high amounts of monounsaturated fatty acids (MUFAs) (65.91 ± 1.75–67.75 ± 1.41%), followed by saturated fatty acids (SUFAs) (20.65 ± 0.38–22.03 ± 1.11%) and polyunsaturated fatty acids (PUFAs) (10.2 ± 0.36–12.53 ± 0.73%) (Table 4). The SUFA content of morphotype 2 was significantly lower than that of morphotypes 1 and 3. Morphotype 3 had significantly lowest PUFA content compared to morphotypes 1 and 2. The MUFA content was not significantly different among the morphotypes. The SUFA, MUFA, and PUFA proportions were similar to those previously reported [9]. However the tigernut studied here had better SUFA and PUFA content than those reported by Sánchez-Zapata et al. [3]. A total of seventeen fatty acids have been identified in each morphotype. Among the fatty acids, oleic acid (64.25 ± 1.99–65.76 ± 1.8%), palmitic acid (15.22 ± 0.55–15.83 ± 0.32%), linoleic acid (10.04 ± 0.37–12.39 ± 0.72%), and stearic acid (3.89 ± 0.15–5.36 ± 0.26%) were the most abundant fatty acids, in three morphotypes, as previously reported [5, 35].

### 3.7. Mineral Contents

The three* Cyperus esculentus* morphotypes tubers appeared to be important sources of mineral (Table 5). The most abundant minerals were K, P, Si, Cl, S, and Mg and their content was significantly different at *p* < 0.05 except for S and Mg. Some contaminants such Cr, Sr, and Cd were detected at low amounts.

Morphotype 1 was found to be richer in Ca, Cu, and Mn contents. Al, Mg, P, S, and Si were most abundant in morphotype 2. Morphotype 3 had the highest content of Cl, K, and Zn. The mineral compositions of the three morphotypes in the present study are different from those recorded with accessions from Niger, Nigeria, and Turkey [22, 35, 36]. Field observations of the soil type where the tigernuts are mainly growing showed that morphotype 1 is grown on more sandy soil, whereas morphotypes 2 and 3 are cultivated on soil with, respectively, more reddish and brownish clay. Therefore different mineral content can be due to differences in soil composition which can influence mineral uptake and storage in the tuber.

### 3.8. Principal Component Analysis

**Figure 2:**
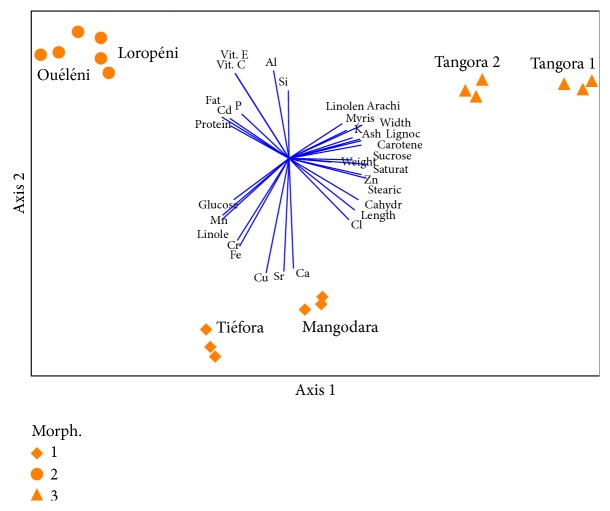
Ordination of the Burkina Faso* Cyperus esculentus* tubers showing three morphotypes. Myris = myristic acid, Stearic = stearic acid, Arachi = arachidic acid, Lignoc = lignoceric acid, Saturat = total saturated acids, Linole = linoleic acid, Linolen = linolenic acid, Cahydr = carbohydrates, Vit. C = vitamin C, and Vit. E = vitamin E.

Principal component analysis (PCA) is used in exploratory analysis, which gives an overview of multivariate data [37]. A PCA using 33 physical and chemical variables showed clear differences among the three morphological types of tubers and gives a good overview of the characteristics of each type (Figure 2).

The first three principal components accounted for 82.27% of the total variation among the accessions. Most of the variation was explained by the first principal component (52%), followed by the second (22%) and the third (9%) (Table 6).

Loadings of the variables on the first two principal components show that the first component had high positive loadings from length, width, carbohydrate, sucrose, *β*-carotene, stearic acid, stearic acid, linoleic acid, total saturated fatty acids, and Zn and high negative loadings from lipids, linolenic acid, and Mn. The second component had high negative loadings from Cu, Sr, and Ca. Morphotype 1 had negative loadings in PC2 and was characterized by high Ca, Sr, Cu, and Fe contents whereas morphotype 2 showed positive scores in PC2 and had high protein, lipid, vitamin C, vitamin E, and P contents. Morphotype 3 was located in the positive side of PC1 and was characterized by the highest content of ash, *β*-carotene, and myristic, arachidic, and linolenic acids.

## 4. Conclusion

We present in this study the physical and chemical variability of tigernuts (*Cyperus esculentus*) cultivated in Burkina Faso. The data revealed that three* Cyperus esculentus* morphotypes are important source of macronutrients (starch, fat, and sucrose) and minerals (potassium, phosphorus, silicon, chlorine, sulfur, and magnesium). Some interesting differences were noticed such as the content of carbohydrates, starch, saturated fatty acids, and polyunsaturated fatty acids. The yellow morphotypes showed the highest content of fructose, glucose, and crude oil. Black morphotype was richer in carbohydrates with high content in sucrose whereas the yellow are source of fructose and glucose. These data revealed genetic variability among cultivated tigernuts from Burkina Faso and from others grown worldwide. Thus, tigernuts from Burkina Faso displayed particular composition which could be of great interest for nutritional quality and food processing.

## Figures and Tables

**Table 1:** Physical characteristics and proximate composition of *Cyperus esculentus* morphotypes tubers.

Parameters	Morphotype 1	Morphotype 2	Morphotype 3
Mean length (cm)	1.24 ± 0.05^a^	0.98 ± 0.06^b^	1.31 ± 0.06^a^
Mean width (cm)	0.97 ± 0.05^a,b^	0.90 ± 0.08^b^	1.19 ± 0.05^a^
1000 dried tubers (g)	814.3 ± 184.1^a^	598.00 ± 115.00^b^	1044.00 ± 394.60^a^
Moisture (g 100 g^−1^)	5.19 ± 0.18^a^	4.56 ± 0.22^a^	4.99 ± 0.78^a^
Crude oil (g 100 g^−1^)	26.14 ± 0.71^b^	28.94 ± 0.37^a^	24.91 ± 0.94^c^
Protein (g 100 g^−1^)	3.47 ± 0.71^a,b^	4.33 ± 0.6^a^	3.3 ± 0.26^b^
Ash (g 100 g^−1^)	1.81 ± 0.24^a,b^	1.69 ± 0.21^b^	2.21 ± 0.39^a^
Carbohydrates (g 100 g^−1^)	68.24 ± 1.28^a,b^	64.73 ± 1.21^b^	69.21 ± 1.30^a^

Values are means ± standard deviation for *n* = 3. Data in the same row followed by different letters are significantly different (*p* < 0.05).

**Table 2:** Carbohydrate composition (g 100 g^−1^) of *Cyperus esculentus *morphotypes tubers.

Parameters	Morpho. 1	Morpho. 2	Morpho. 3
Starch	30.54 ± 2.75^a^	33.21 ± 1.1^a^	30.54 ± 0.5^a^
Sucrose	18.99 ± 0.56^b^	17.98 ± 1.03^b^	20.39 ± 1.15^a^
Fructose	3.02 ± 0.37^a^	3.59 ± 0.72^a^	1.6 ± 0.69^a^
Glucose	6.79 ± 1.34^a^	6.33 ± 0.97^a^	0 ± 0^b^

Values are means ± standard deviation for n = 3. Data in the same row followed by different letters are significantly different (*p* < 0.05).

**Table 3:** Vitamin contents of *Cyperus esculentus* morphotypes tubers.

Parameters	Morph. 1	Morph. 2	Morph. 3
Vitamin C (mg 100 g^−1^)	5.48 ± 1.05^c^	26.78 ± 2.51^a^	8.33 ± 1.83^b^
Vitamin E (*µ*g 100 g^−1^)	209.71 ± 1.30^b^	270.56 ± 1.74^a^	149.86 ± 1.94^c^
*β*-Carotene (*µ*g 100 g^−1^)	7.3 ± 0.57^b^	6.13 ± 0.62^c^	10.05 ± 1.79^a^

Values are means ± standard deviation for n = 3. Data in the same row followed by different letters are significantly different (*p* < 0.05).

**Table 4:** Fatty acid composition (% total fatty acids) of total lipid from *Cyperus esculentus* morphotypes tubers' oil.

Fatty acids	Morphotype 1	Morphotype 2	Morphotype 3
Myristic acid	0.16 ± 0.03^b^	0.14 ± 0^b^	0.4 ± 0.24^a^
Pentadecanoic acid	0.03 ± 0.03^a^	0 ± 0^a^	0.05 ± 0.05^a^
Palmitic acid	15.81 ± 0.95^a^	15.83 ± 0.32^a^	15.22 ± 0.55^a^
Stearic acid	4.73 ± 0.33^b^	3.89 ± 0.15^c^	5.36 ± 0.26^a^
Arachidic acid	0.57 ± 0.03^b^	0.55 ± 0.02^b^	0.68 ± 0.01^a^
Behenic acid	0.1 ± 0^a^	0.09 ± 0.02^a^	0.1 ± 0.01^a^
Lignoceric acid	0.17 ± 0.01^b^	0.15 ± 0.03^b^	0.24 ± 0.02^a^
Palmitoleic acid	0.40 ± 0.05^a^	0.37 ± 0.04^a^	0.53 ± 0.02^a^
*cis*-7-Hexadecenoic acid	0.35 ± 0.05^a^	0.33 ± 0.03^a^	0.44 ± 0.2^a^
Heptadecenoic acid	0 ± 0^a^	0.03 ± 0.03^a^	0.03 ± 0.03^a^
Oleic acid	64.25 ± 1.99^a^	65.42 ± 1.17^a^	65.76 ± 1.8^a^
Vaccenic acid	0.99 ± 0.14^a^	0.99 ± 0.12^a^	0.93 ± 0.11^a^
Eicosenoic acid	0.25 ± 0.05^a^	0.28 ± 0.06^a^	0.37 ± 0.16^a^
Cetoleic acid	0.05 ± 0.05^a^	0.03 ± 0.03^a^	0.09 ± 0.1^a^
Nervonic Acid	0 ± 0^a^	0.05 ± 0.05^a^	0.05 ± 0.05^a^
Linoleic acid	12.39 ± 0.72^a^	12.07 ± 0.23^a^	10.04 ± 0.37^b^
Linolenic acid	0.14 ± 0.02^b^	0.14 ± 0.01^b^	0.17 ± 0.01^a^
Total saturated	21.56 ± 0.71^a^	20.65 ± 0.38^b^	22.03 ± 1.11^a^
Total monounsaturated	65.91 ± 1.75^a^	67.15 ± 1.21^a^	67.75 ± 1.41^a^
Total n-6 PUFA	12.39 ± 0.72^a^	12.07 ± 0.23^a^	10.04 ± 0.37^b^
Total n-3 PUFA	0.14 ± 0.02^b^	0.14 ± 0.01^b^	0.17 ± 0.01^a^
Total PUFA	12.53 ± 0.73^a^	12.21 ± 0.23^a^	10.2 ± 0.36^b^

Values are means ± standard deviation for *n* = 3. Data in the same row followed by different letters are significantly different (*p* < 0.05).

**Table 5:** Mineral composition (mg 100 g^−1^) of *Cyperus esculentus* morphotypes tubers.

Parameters	Morph. 1	Morph. 2	Morph. 3
Al	34.35 ± 2.55^b^	43.37 ± 6.13^a^	39.86 ± 0.74^a^
Ca	32.27 ± 5.66^a^	19.09 ± 3.22^b^	22.13 ± 1.64^b^
Cd	0.01 ± 0.01^a,b^	0.02 ± 0.01^a^	0.01 ± 0^b^
Cl	167 ± 0.53^a^	155.4 ± 2.83^b^	167.2 ± 5.51^a^
Cr	1.65 ± 0.05^a^	1.12 ± 0.31^b^	0.18 ± 0.01^c^
Cu	0.71 ± 0.03^a^	0.48 ± 0.05^b^	0.43 ± 0.01^c^
Fe	11.44 ± 0.48^a^	8.25 ± 1^b^	3.57 ± 0.17^c^
K	608.3 ± 97.84^b^	556.9 ± 80.4^b^	845.8 ± 7.94^a^
Mg	100.5 ± 1.79^a^	107.3 ± 8.2^a^	102.2 ± 2.86^a^
Mn	1.55 ± 0.44^a^	1.44 ± 0.15^a^	0.38 ± 0.02^b^
P	229.6 ± 51.54^a,b^	283.7 ± 14.96^a^	236.4 ± 16.53^b^
S	164.3 ± 18.23^a^	194.1 ± 61.62^a^	148.8 ± 8.39^a^
Si	181.6 ± 50.35^b^	242.5 ± 35.89^a^	220.3 ± 47.32^a,b^
Sr	0.36 ± 0.09^a^	0.17 ± 0.02^b^	0.19 ± 0.02^b^
Zn	2.34 ± 0.31^b^	1.88 ± 0.22^b^	2.7 ± 0.03^a^

Values are means ± standard deviation for *n* = 3. Data in the same row followed by different letters are significantly different (*p* < 0.05).

**Table 6:** Eigenvectors and percent explained variation by the first six principal components of physical and chemical data parameters of 3 morphotypes of *Cyperus esculentus* tubers.

Variable	Eigenvectors					
	PC1	PC2	PC3	PC4	PC5	PC6
Length	0.201	−0.158	−0.059	0.210	−0.061	0.133
Width	0.217	0.060	−0.044	0.260	−0.092	−0.045
Weight	0.128	−0.012	0.092	0.067	−0.561	0.313
Crude oil	−0.205	0.126	0.207	−0.036	0.067	0.013
Protein	−0.178	0.099	−0.369	−0.073	−0.111	0.050
Ash	0.164	0.042	−0.013	0.147	0.127	0.625
Carbohydrates	0.212	−0.126	0.148	0.053	0.121	0.039
Sucrose	0.195	−0.005	−0.036	0.044	0.092	−0.399
Glucose	−0.170	−0.125	−0.342	−0.206	0.015	0.095
Vitamin C	−0.165	0.259	0.079	0.011	0.166	0.008
Carotene	0.221	0.041	−0.082	−0.185	0.214	0.007
Vitamin E	−0.165	0.259	0.079	0.011	0.166	0.008
P	−0.144	0.135	−0.277	0.407	−0.045	0.009
Cr	−0.158	−0.251	0.197	0.088	0.126	−0.017
Fe	−0.151	−0.268	0.185	0.008	0.123	−0.018
Mn	−0.202	−0.175	−0.067	0.176	0.150	−0.006
Cu	−0.070	−0.349	−0.040	−0.144	0.021	0.065
Zn	0.222	−0.050	0.223	0.048	−0.077	−0.073
Sr	−0.016	−0.344	−0.172	0.194	0.101	0.010
Cd	−0.180	0.121	−0.311	−0.218	0.003	0.061
Ca	0.015	−0.335	−0.135	0.256	0.135	0.007
K	0.194	0.063	−0.191	0.336	−0.051	−0.122
Al	−0.046	0.269	0.068	0.331	0.093	−0.281
Si	−0.003	0.207	0.149	0.132	0.382	0.453
Cl	0.182	−0.186	−0.104	−0.191	0.210	−0.002
Myristic acid	0.177	0.087	−0.156	−0.050	0.450	−0.043
Stearic acid	0.237	−0.062	0.073	−0.038	0.043	−0.042
Arachidic acid	0.224	0.100	−0.016	−0.192	−0.079	−0.007
Lignoc	0.220	0.054	−0.133	−0.252	−0.005	0.017
Saturat	0.239	−0.019	0.015	−0.065	0.117	−0.040
Linole	−0.206	−0.187	−0.067	0.056	0.006	0.010
Linolen	0.164	0.105	−0.414	0.093	0.024	0.025
Eigenvalue	16.69	6.97	2.67	1.81	1.71	0.79
Individual %	52.14	21.78	8.34	5.66	5.33	2.46
Cumulative %	52.14	73.93	82.27	87.92	93.26	95.72
